# Patterns, Risks, and Clinical Challenges of Pregabalin Misuse in Patients With Opioid Use Disorder: A Case Series

**DOI:** 10.7759/cureus.84524

**Published:** 2025-05-21

**Authors:** Nikhil Gurjar, Tanu Singla, Nikhil Gautam

**Affiliations:** 1 Psychiatry, Gurjars Clinic, Mumbai, IND; 2 Psychiatry, Civil Hospital Tapa, Barnala, IND; 3 Psychiatry and Behavioral Sciences, Christian Medical College and Hospital, Ludhiana, IND

**Keywords:** case series, opioid use disorder, polysubstance use, pregabalin abuse, withdrawal

## Abstract

Pregabalin, a gabapentinoid used for neuropathic pain and epilepsy, is increasingly misused, particularly among individuals with opioid use disorder (OUD). Its synergistic effects with opioids enhance euphoric sensation. Pregabalin misuse in OUD patients is driven by this enhanced euphoria and self-medication to control opioid withdrawal symptoms, and pregabalin is used as an opioid substitution. This increases the risk of respiratory depression and leads to severe withdrawal symptoms. This case series presents three individuals with comorbid OUD and high-dose pregabalin dependence, detailing their withdrawal symptoms, co-occurring substance use, and treatment intervention. Stricter regulations, prescriber awareness, and harm reduction strategies are essential to prevent such misuse.

## Introduction

Pregabalin is a molecule that is structurally similar to gamma-aminobutyric acid and belongs to a class of drugs known as gabapentinoids. It is an agent approved for use in neuropathic pain associated with spinal cord injury, diabetes-related peripheral neuropathy, and herpes-related neuralgia. It is also indicated for adjunctive use in partial-onset seizures in patients with epilepsy [1]. It is also indicated for use in fibromyalgia. However, it is used off-label by patients suffering from anxiety disorders, insomnia, and other psychiatric disorders [2].

Unfortunately, it has also become a drug that is now frequently being misused by patients in India and other countries [3,4]. In Punjab, a state in northern India, increased use and seizures of large quantities of drugs by law enforcement led to the administration of the region to prohibit the sale of pregabalin tablets beyond 75mg strength without a prescription [5].

Despite its therapeutic benefits, reports of pregabalin misuse have been steadily rising, often in populations already vulnerable to substance-related harms. Among these populations, individuals with opioid use disorder (OUD) are at heightened risk due to their predisposition for polysubstance use and the synergistic effects of pregabalin when combined with opioids. This combination not only amplifies euphoric sensations but also significantly increases the risk of respiratory depression and other adverse outcomes [6,7].

This paper presents a case series highlighting instances of pregabalin abuse among patients diagnosed with OUD. By examining the clinical presentations, patterns of misuse, and associated outcomes, we aim to deepen the understanding of this emerging public health issue. The focus is on the need for heightened vigilance among healthcare providers for OUD patients coming with very high doses of pregabalin abuse as well as the development of targeted interventions to mitigate the risks associated with pregabalin misuse in this vulnerable population.

## Case presentation

Case 1

A 28-year-old man, employed as a farmer, was brought to the emergency department after experiencing a seizure episode. The event was characterized by loss of consciousness, abnormal body movements, upward rolling of the eyes, clenching of teeth, tongue biting, and confusion after the seizure, all of which were indicative of generalized seizures. Upon detailed inquiry, it was revealed that the patient had been consuming pregabalin at a very high dose, 20 tablets of 300 mg strength daily, for the past 1.5 years. Additionally, he reported intermittent use of poppy husks, which are field-dried leaves, stalks, and seed pods derived from opium poppies when pregabalin was unavailable.

The informant noted that the patient had discontinued pregabalin abruptly two days prior due to unavailability and had experienced a similar seizure episode the day before presentation. Upon arrival at the hospital, he was treated with an intravenous injection of diazepam (5 mg) to control the seizure activity.

Following initial stabilization, the patient was started on intravenous diazepam (5 mg twice daily), oral oxcarbazepine (300 mg twice daily), and tapentadol (100 mg thrice daily) to manage withdrawal symptoms and prevent further seizures. Routine blood tests, including viral markers, revealed hepatitis C positivity, for which he had no prior treatment history.

Over the next three days, his condition stabilized, and he was discharged in satisfactory condition on oral medications: oxcarbazepine (300 mg twice daily), tapentadol (100 mg thrice daily), and lorazepam (1 mg in the morning and 2 mg at night). At his one-week follow-up, the patient reported no recurrence of seizures, and his overall condition had improved significantly. This case shows the risks of abrupt withdrawal from pregabalin in individuals with a history of high-dose use, especially when coupled with other substances like poppy husk (Table 1).

**Table 1:** Summary of substance use pattern, symptoms and treatment used in patients

Patient case number	Age of patient (in years)	Number of tablets and dosage of pregabalin consumed daily	Duration of pregabalin misuse	Withdrawal symptoms experienced	Treatment received
1	28	20 tablets/daily (6000mg/daily)	1 year and 6 months	Generalized seizures	Oxcarbazepine (300 mg twice daily), tapentadol (100 mg thrice daily), lorazepam (1 mg in the morning and 2 mg at night)
2	30	15-20 tablets (4500-6000mg/daily)	1 year	Myoclonus, generalized body aches, sleep disturbance, irritability	Tapentadol (100 mg three times daily), clonidine (0.1 mg twice daily), lorazepam (2 mg at night)
3	35	15-20 tablets (4500-6000mg/daily)	2 years	Low mood, anxiety, restlessness, generalized body aches, sleep disturbance	Gabapentin (300 mg) with nortriptyline (10 mg twice daily), flupirtine (400 mg once daily), paracetamol (325 mg) with aceclofenac (100 mg twice daily), sertraline (25 mg twice daily), clonazepam (2 mg at night)

Case 2

A 30-year-old married male farmer presented to the psychiatric outpatient department with complaints of physical and psychological symptoms that had arisen after discontinuing pregabalin use. The patient disclosed a history of consuming 15-20 pregabalin tablets daily for the past year. Prior to this, he had been consuming poppy husk at a substantial amount of 3-4 kg per month for approximately five years before transitioning to pregabalin.

He reported that for the past week, he had been unable to procure pregabalin, which led to the onset of withdrawal symptoms. These included sudden, involuntary jerky movements of the body that even occurred during sleep, generalized body aches, increased irritability, and significant sleep disturbances.

To address his withdrawal symptoms, the patient was started on a medication regimen comprising tapentadol (100 mg three times daily) for pain relief and withdrawal management, clonidine (0.1 mg twice daily) to alleviate autonomic symptoms, and lorazepam (2 mg at night) to help with sleep disturbances and anxiety.

By his follow-up visit after five days, the patient reported significant improvement in his symptoms. The frequency and intensity of the jerky movements had markedly reduced, his body aches had resolved, and his sleep pattern had normalized. This case highlights the challenges of pregabalin withdrawal and its management in individuals with a history of opioid use to support recovery (Table 1).

Case 3

A 35-year-old married male barber sought treatment at the psychiatric outpatient department for persistent low mood, anxiety, restlessness, and disturbed sleep. On further inquiry, it was revealed that the patient had been consuming 15-20 pregabalin tablets daily for the past two years, along with 20-30 chewable tobacco pellets daily for five years. Additionally, he consumed alcohol occasionally, approximately 180-250 ml, two to three times per month. His past history was suggestive of consumption of opium 2 to 3 grams for three years, before slowly shifting over to pregabalin.

Due to financial difficulties, the patient expressed a desire to quit all his substance use. He reported having attempted to stop pregabalin multiple times on his own but experienced withdrawal symptoms, including generalized body aches, diarrhea, and severe sleep disturbances, which compelled him to resume its use.

Routine laboratory investigations, including blood tests, thyroid function tests, and viral markers, were conducted and found to be within normal limits. The patient was started on a comprehensive treatment plan to manage both his withdrawal symptoms and co-occurring psychiatric issues. This included a combination of gabapentin (300 mg) and nortriptyline (10 mg) twice daily for neuropathic pain and mood stabilization, flupirtine (400 mg once daily) as an analgesic, and paracetamol (325 mg) with aceclofenac (100 mg) twice daily for pain relief. Additionally, he was prescribed sertraline (25 mg twice daily) for his low mood and clonazepam (2 mg at night) for his sleep disturbances and anxiety.

To address his tobacco dependence, nicotine replacement therapy in the form of 4 mg nicotine gum was initiated, with instructions to use it three times daily. The patient was also counseled on the risks of alcohol use and advised to abstain.

At his one-week follow-up, the patient reported significant progress. He had successfully discontinued pregabalin without any adverse events and reported improvement in mood, sleep quality, and overall well-being (Table 1).

## Discussion

This case series presents three distinct instances of pregabalin misuse among patients with OUD, highlighting the clinical challenges and significant withdrawal symptoms associated with abrupt cessation of high-dose pregabalin. Pregabalin is widely recognized for its therapeutic benefits in neuropathic pain and anxiety-related disorders, but its misuse is becoming increasingly prevalent, particularly in regions where opioid use is already a public health concern [8]. These cases underscore the severe withdrawal symptoms associated with high-dose pregabalin dependence, including seizures, involuntary jerky movements, body aches, psychological distress, and sleep disturbances. These withdrawal effects present clinical challenges and often require careful pharmacological management, including gradual dose tapering, symptomatic treatment, and addressing co-occurring substance use. The risks associated with pregabalin misuse in vulnerable populations reveal patterns of dependency, withdrawal symptoms, and co-occurring substance use that complicate management and recovery.

It should also be noted that these cases signify that pregabalin abuse is not an isolated phenomenon but rather a complex issue driven by its synergistic effects with opioids, self-medication for withdrawal symptoms, accessibility, and substitution for illicit opioids.

A possible explanation is that gabapentinoids appear to amplify opioid effects, increasing analgesia and overdose-related adverse outcomes, the underlying mechanism apparently being gabapentinoid potentiation of opioid associated effects on cyclic AMP signaling in neuropathic pain [9]. Furthermore, the target of pregabalin is the α2-δ1 auxiliary subunits of voltage-dependent calcium channels, while the target of opioids is μ-opioid receptors. Both activate the descending noradrenergic system, thus affecting pain reception [10]. Interestingly, pregabalin appears not to be metabolized and has no effect on the functional expression of cytochrome P450 isozymes. Hence, it is considered that there is no or only a negligible possibility of a pharmacokinetic interaction between pregabalin and opioids [11]. 

Previous research, however, has noted that incidences of the adverse effects in patients who used pregabalin and opioids concomitantly were overall higher than those who used pregabalin without opioids, citing that it might be caused by the additive effect of opioids [12]. The synergistic action between opioids and gabapentinoids has been correlated with increased dopaminergic activity, which may intensify euphoria [13].

Due to the sedative effects of pregabalin, many patients use it to ease their substance-associated withdrawal symptoms, such as restlessness and anxiety, and also achieve sedation [14]. Thus, it may serve as a ‘substitute’ when illicit opioids are unavailable due to legal, financial, or personal constraints. It is an easily available medication in most pharmacies, and easy access can increase the risk of abuse. Persons with illicit opioid use may consider it a ‘safer’ alternative to their drug of choice. Inadequate prescription monitoring practices and sale of drugs without prescription are frequently seen problems in India, which further contributes to the risk of increased misuse [15].

These findings are reflected in current cases as well, where patients had a previous history of opioid use, had easy access to the drug without the necessary supervision, a tendency to take more than the advised dosage, and misusing pregabalin in a bid to use it as a substitute for opioids.

The authors acknowledge that the presented case series reveals just the tip of the iceberg with regard to problems associated with pregabalin misuse in OUD. Severity of withdrawal symptoms was not assessed on any objective rating scale, and noted findings were based solely on the report of the patients. Furthermore, the findings were localized to a specific location of the Punjab region in Northern India and do not capture the full picture of misuse practices done by patients of OUD elsewhere. Future research is warranted to better understand the long-term consequences of pregabalin misuse in OUD populations, optimal treatment strategies, and the effectiveness of policy regulations in reducing its non-medical use.

From a clinical perspective, early identification of pregabalin misuse, screening in high-risk populations (such as individuals with OUD), and implementing comprehensive harm reduction strategies are essential to mitigate the risks. Alternative strategies can be debated as patterns of substance abuse disorders and management of drugs may differ from country to country in accordance with government policies. A multidisciplinary approach, incorporating pharmacological treatment, psychosocial interventions, and long-term monitoring, will thus be crucial for effectively managing pregabalin dependence and preventing relapse.

## Conclusions

This case series highlights the emerging issue of pregabalin misuse among individuals with OUD, emphasizing the significant risks associated with its high-dose consumption, abrupt discontinuation, and polysubstance use. The sale of high-dose pregabalin without proper medical supervision poses a significant public health risk, necessitating stricter prescription monitoring, awareness programs, and policy interventions to prevent misuse. Addressing this growing concern thus requires collaborative efforts from healthcare professionals, policymakers, and public health authorities to enhance patient safety and minimize the harms associated with pregabalin misuse.
